# Utilization of Psychotherapeutic Interventions by Pediatric Psychosocial Providers

**DOI:** 10.3390/children8111045

**Published:** 2021-11-12

**Authors:** Cynthia Fair, Amanda Thompson, Marie Barnett, Stacy Flowers, June Burke, Lori Wiener

**Affiliations:** 1 Department of Public Health Studies, Elon University, Elon, NC 27244, USA; jburke13@elon.edu; 2 Inova Schar Cancer Institute, Fairfax, VA 22031, USA; Amanda.Thompson@inova.org; 3 Memorial Sloan Kettering Cancer Center, New York, NY 10065, USA; barnettm@mskcc.org; 4 Department of Family Medicine, Boonshoft School of Medicine, Wright State University, Dayton, OH 45406, USA; stacy.flowers@wright.edu; 5 Pediatric Oncology Branch, Center for Cancer Research, National Cancer Institute, National Institutes of Health, Bethesda, MD 20892, USA; wienerl@mail.nih.gov

**Keywords:** standards of psychosocial care for children with cancer and their families, psychotherapeutic interventions, evidence-based interventions, providers, psycho-oncology

## Abstract

One of the Standards of Psychosocial Care for Children with Cancer and their Families recommends that all youth with cancer and their family members have access to psychotherapeutic interventions and support throughout the cancer trajectory. This study was created to identify the psychosocial interventions and services provided to children with cancer and their family members, to ascertain whether there are differences in interventions provided by age of the patient and stage of treatment, and to learn about barriers to psychosocial service provision. An online survey was disseminated to psychosocial providers through the listservs of national and international professional organizations. The majority of the 242 respondents were either psychologists (39.3%) or social workers (26.9%) and 79.7% worked in the United States. The intervention offered most often to pediatric patients, caregivers, and siblings, at every stage of treatment, was psychoeducation (41.7–48.8%). Evidence-based interventions, including cognitive behavioral therapy (56.6%) and mindfulness-based interventions (57.9%) were reported to be frequently used with patients. Interventions designed specifically for the pediatric oncology population were not commonly endorsed. Psychosocial providers reported quality of care would be improved by additional staff, better communication/collaboration with medical team members and increased community-based resources. Future research should focus on improving accessibility to population-specific evidenced-based interventions and translating science to practice.

## 1. Introduction

Children with cancer and their families experience various psychosocial challenges during and after treatment, including physical and cognitive changes due to medical treatment, alterations in social and familial roles, and the potential threat of death [1]. Most children and families cope and adjust well to the diagnosis and treatment of cancer [2]. However, there is a subset of children with cancer at increased risk for anxiety and depression, as well as educational and relationship difficulties [1,3]. Caregivers and siblings of pediatric patients are at risk for heightened distress and post-traumatic stress [4,5]. Psychosocial care and interventions can play an important role in supporting the well-being of the entire family, beginning at diagnosis [6]. 

The Standards of Psychosocial Care for Children with Cancer and their Families [7] recommend that “youth with cancer and their family members should have access to psychosocial support and interventions throughout the cancer trajectory and access to psychiatry as needed” (p. S585) [3]. Currently, there are only a few evidence-based psychotherapeutic interventions designed to decrease psychological distress and increase emotional well-being specifically for the pediatric oncology population [8,9]. These include the Surviving Cancer Competently Intervention Program (SCCIP) and Bright IDEAS Problem Solving Skills Training, two programs targeting distress in parents of children with cancer, demonstrating high degrees of effectiveness, and designated as evidence-based cancer control programs (EBCCP) by the National Cancer Institute (NCI). Alternatively, a range of additional interventions exist, not specifically adapted for or tested with oncology populations. These include psychoeducation, supportive individual therapy, cognitive behavioral therapy, problem-solving therapy, social support groups, social skills training, health promotion, bibliotherapy, art therapy, and mindfulness-based therapies [10,11]. Yet, we currently do not understand what clinical interventions are being offered by psychosocial providers or nuanced differences among provider specialties and professional stage, and it is unlikely that all pediatric cancer centers have the resources to offer clinical training and the full array of evidence-based interventions [7]. There is some evidence that professional stage may impact care. Ramsmussen and colleagues found that older psychosocial oncologists reported significantly less burnout than their younger counterparts [12]_._

This study was designed to learn how pediatric cancer treatment programs currently address the Psychosocial Standard to provide psychotherapeutic interventions. We aimed to describe the psychotherapeutic interventions that psychosocial providers are using “in the real world” throughout the cancer trajectory (i.e., initial diagnosis, on treatment, survivorship, end of life), explore potential group differences by provider type (i.e., psychologist, social worker, child life specialist, etc.) and professional stage (i.e., early-, mid-, late-career), and to identify the education and resource needs of pediatric psychosocial providers. 

## 2. Materials and Methods

### 2.1. Survey Development

A survey was created by the authors, representing pediatric psychosocial oncology providers (CF, AT, MB, and LW), with a cumulative 72 years of experience in psychosocial oncology. The survey consisted of practitioner demographic information including discipline, education, and professional career stage. Institutional type and location, information related to number of new pediatric oncology patients seen per year, and number of psychosocial providers on staff were collected. The survey took approximately 15–20 min to complete. 

In a quantitative format, questions assessed current interventions and services available to pediatric cancer patients and their families. The list of intervention types was developed and categorized based on a review of relevant literature and the combined experience of the authors. Survey items were reviewed by the group during monthly meetings and verbal consensus was reached on questions prior to the launch. Categories were not mutually exclusive but, instead, allowed for an increasing degree of specificity (e.g., CBT, mindfulness-based interventions, and problem-solving therapies were listed separately, despite the fact that mindfulness and problem-solving are recognized as types or components of CBT). The survey also asked about the age of patients, the population to whom interventions were offered (patient, caregiver, siblings), and the frequency and timing of when interventions were offered throughout the cancer trajectory. 

In a qualitative format, psychosocial providers were asked what they perceived was needed to better serve pediatric cancer patients and their families. Through open-ended response options, respondents had the chance to note challenges to providing care (please see survey in Supplementary File S1). Research was approved by Elon University’s Institutional Review Board (#17-184) on 26 March 2019.

### 2.2. Recruitment Procedures

In May 2019, the survey was disseminated online through Qualtrics to psychosocial providers through three professional organization listservs: American Psychosocial Oncology Society (APOS), Association of Pediatric Oncology Social Workers (APOSW), and the Hematology/Oncology/Bone Marrow Transplant Special Interest Group through the Society of Pediatric Psychology (SPP), which offers membership to providers in the United States, Canada and abroad. Respondents were able to share the link with other members in their institution regardless of membership in the listed organizations. A reminder email to complete the survey was sent out two weeks later. The survey closed July 2019.

### 2.3. Data Analysis

#### 2.3.1. Quantitative Analysis

All quantitative data were downloaded from Qualtrics into Excel. Univariate analyses were conducted to describe the frequency and timing of identified psychotherapeutic interventions, as well as which populations (patients, siblings, or caregivers) were provided the interventions. Additional analyses characterized how different providers engaged in providing psychosocial care to pediatric cancer patients and their families. Bivariate analyses explored whether certain interventions are more likely to be used with pediatric cancer patients of a particular age or during a specific stage of treatment. 

#### 2.3.2. Qualitative Analysis

Responses to the question “Name three things that would improve your ability to do your job better” were coded by two co-authors (JB and CF) using a content analysis approach [13]. Coders independently reviewed responses, developed categories, and met frequently to discuss identified themes, coming to consensus on categories [14].

## 3. Results

### 3.1. Demographics

Two hundred forty-two psychosocial providers responded to the survey (Table 1). The majority were either psychologists (39.3%) or social workers (26.9%) and identified as either early (36.8%) or mid-career (32.2%) licensed professionals. Seventy-nine percent worked in the United States, with most working at a pediatric-specific hospital (57.4%) or an academic medical center (47.1%); 74.4% provided inpatient services and 68.2% provided outpatient services. The number of new pediatric oncology patients seen yearly at respondents’ institutions varied (M = 188.6, range 12–650) as did the number of psychosocial providers (Table 1).

### 3.2. Treatment Population

Approximately half of respondents’ practice involved treating patients between ages 13 and 17 (44.5%), and over a third treated patients between ages 0 and 12 (38.4%). Twenty percent of respondents indicated that caregivers comprised 5–24% of their treatment population. Siblings were included less often, as 44.6% of respondents noted siblings comprised <5% of their practice (Table 2).

### 3.3. Types of Interventions Offered

The interventions most often offered were psychoeducation (48.8%), mindfulness-based interventions (44.2%), anticipatory guidance (42.1%), cognitive behavioral therapy (CBT) (42.1%), health promotion interventions (42.1%), supportive individual psychotherapy (38.9%), and referrals to social support groups (38.0%). Interventions changed across the cancer trajectory with psychoeducation being used most often at initial diagnosis, during active treatment, during maintenance, in survivorship and at relapse. Supportive individual psychotherapy was the most used intervention at end of life and bereavement. Anticipatory guidance was the second most used intervention at initial diagnosis and at end of life. Respondents were more likely to provide psychotherapeutic interventions during active treatment and initial diagnosis and least likely to provide interventions at end of life and bereavement along the cancer trajectory. Cancer-specific interventions such as SCCIP and Bright Ideas were offered, although less frequently (Table 3). More than one response was allowed for this question. 

**Figure 1:**
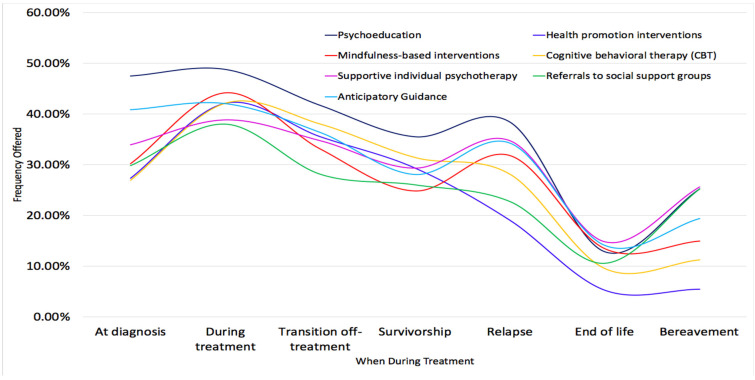
Interventions Offered Throughout Cancer Trajectory.

Figure 1 offers an alternative way of viewing interventions offered across the cancer trajectory. It highlights the prominence of psychoeducation over time. The ebb and flow of when interventions are offered is evident with increases during treatment, at relapse and bereavement, as well as the general downward trajectory with fewer interventions offered as treatment progresses. 

### 3.4. Group Differences

There were no notable differences between type of provider or professional stage and the type of psychotherapeutic interventions provided for children on active treatment. The intervention most commonly offered to children undergoing cancer treatment was psychoeducation (66.1%), followed by health promotion interventions (e.g., nutrition, physical activity, sleep) (59.9%), mindfulness-based interventions (57.9%), cognitive behavioral therapy (56.6%), and supportive individual therapy (54.5%). Interventions offered to caregivers and siblings are presented in Table 4. Respondents were asked to check all that apply.

Psychosocial providers also recommended apps to patients and families, most commonly apps for breathing/relaxation (53.7%), meditation (36.4%), and guided imagery (32.2%) (Table 5). 

### 3.5. Educational and Resource Needs of Psychosocial Providers

#### 3.5.1. Staff Resources

Respondents described needed resources that would help them provide better psychosocial care. Additional staff, more time to dedicate to providing direct care, and additional trainings, including clinical supervision, were most often reported as needed in order to provide better psychosocial care. As one respondent stated, “We need more time, more staff, and more resources to provide services.” Psychiatric services were described as important but difficult to access. One respondent suggested “(we need) better availability of psychiatrists to reduce the wait time for outpatient services.” Another respondent spoke specifically to the lack of education, “A greater availability of continuing education for psychosocial providers within the oncology arena of conferences and meetings rather than seeking this separately from psychiatry and mental health professional development”. Table 6 includes themes and additional illustrative quotes.

#### 3.5.2. Communication and Collaboration

Respondents also noted the need for improved communication and collaboration across disciplines. A desire to communicate the importance of the Psychosocial Standards more effectively to team members as well as strategies for implementing the Standards was identified. Some respondents sought ways to increase understanding of the role that psychosocial providers play in the care of pediatric cancer patients and their families through advocacy. 

#### 3.5.3. Family Resources

A need for additional family resources was noted, specifically those addressing the mental health needs of caregivers and families, and those that can be offered in the community. For example, one respondent commented, “I wish we could keep an up-to-date list of community mental health resources so families could receive care in their local area. However, it’s hard when families come from many different places to receive care at our center.” Additional respondents wanted family-friendly resources that explained psychosocial services to families. One respondent suggested “I’d like a short guide to help parents cope at diagnosis like a generic version of the Psychosocial Standards of Care.”

## 4. Discussion

This paper describes the psychotherapeutic interventions provided to pediatric cancer patients and their families throughout the cancer trajectory. Most respondents were psychologists or social workers, the majority of whom were early- or mid-career providers. Findings underscore the importance of offering services to patients and caregivers and the dearth of psychosocial care being provided to siblings [5,15]. Lack of group differences between type of provider or professional stage and type of psychotherapeutic interventions being utilized may speak to the type and availability of training across settings and disciplines. It is important that psychosocial providers have the credentialing and training necessary to offer specialized interventions. Results are consistent with prior research demonstrating limited access to and availability of psychiatrists on psychosocial teams [16].

We found that a broad range of psychotherapeutic interventions are being offered to children undergoing cancer therapy and their families. Psychoeducation, defined broadly as the incorporation of illness-specific information and education synergized with strategies for psychotherapeutic management [17,18], was a foundational intervention, and the most frequently endorsed intervention provided to children, caregivers, and siblings. As a distinct Standard of psychosocial care, experts recommend that youth with cancer and their family members receive psychoeducation and anticipatory guidance related to disease, treatment, acute and long-term effects, hospitalization, procedures, and psychosocial adaptation [19]. Psychoeducation is most effective when content is catered specifically to the patient’s needs and provided throughout the trajectory of care. The nature of the psychoeducational information shared was not covered by the current study. However, our findings indicate it is consistently offered throughout the cancer trajectory. Not only can psychoeducation help meet unmet information needs, but it has been shown to improve disease-related knowledge and increase the health locus of control [6,13]. 

Psychosocial providers offered referrals to support groups, cognitive behavioral therapy (CBT), mindfulness, and supportive individual psychotherapy, demonstrating that “one size does not fit all”. Tailored interventions are based on many factors, requiring clinical skills to determine the appropriate interventions, which can vary by medical treatments, psychosocial factors, and availability/access to providers throughout treatment. Further, providers are utilizing multiple interventions, as they are not mutually exclusive. Aspects of mindfulness-based interventions may be offered or utilized during individual supportive psychotherapy or support groups, or individual supportive therapy may utilize mindfulness or psychoeducation in order to establish a trusting relationship between the patient and the care provider [20]. 

Evidence for these diverse approaches within pediatric oncology exists. Research suggests support groups are beneficial to pediatric oncology patients, caregivers, and siblings as they provide individuals with a sense of community [20], and CBT, widely practiced in the field of pediatric oncology, has been found to be effective in lowering symptoms of depression and anxiety and improving levels of self-esteem among patients [21]. Mindfulness, a feasible, effective intervention for adolescents with cancer that is easily adaptable to online formats, has been growing in popularity [22,23]. Psychosocial providers are commonly recommending apps that focus on aspects of mindfulness (e.g., breathing or relaxation techniques, meditation, and guided imagery); however, limited research has explored the effectiveness of readily available apps. This remains an important area for future investigation.

Interestingly, pediatric cancer-specific interventions such as Surviving Cancer Competently Intervention Program [8,24] and Bright IDEAS Problem-Solving Skills Training [9,25,26] were not commonly offered by psychosocial providers, despite robust evidence supporting their efficacy. It is possible that cancer centers may not have access to the necessary resources (e.g., funding additional staff, or provider trainings) to offer these interventions or prioritize their use. Future implementation and dissemination efforts should be made to translate research to practice, increasing access to these interventions for patients and families [27]. This may include addressing implementation barriers by advocating for increased funding for more psychosocial staff, resources, trainings, and overall prioritization of psychosocial services (e.g., messaging from medical providers/institutions, workflow consistency, care coordination among multidisciplinary teams). 

The data suggest that the most commonly offered interventions are provided while the patient is in active treatment. The Psychosocial Standards of care highlight the importance of support at diagnosis and during the early months of treatment, with less frequent delivery of services as the child progresses through treatment [28]. However, patients and families experience increased distress at critical points such recurrence or transition off treatment and, therefore, need additional support at those times as well. It is likely that patients and families have more direct access to psychosocial providers during treatment in both outpatient and inpatient settings. Once children finish cancer treatment, their access to ongoing support from clinic or hospital staff declines as they have less scheduled contacts with providers; additionally, patients may wish to avoid or not return to the hospital whenever possible. As there are abundant data to support the need for psychotherapeutic interventions after cancer therapy ends [29,30], future research should explore the type and availability of interventions later in the cancer trajectory, such as the transition off-treatment and into early and long-term survivorship, and resources needed (e.g., personnel) to provide them. 

Psychosocial providers reported a primary need for additional staff in order to provide quality psychosocial care. Funding for psychosocial positions and inadequate staffing are known and prominent barriers to providing adequate psychosocial care [12,31]. Even in large pediatric oncology programs with greater access to psychosocial care, psychosocial teams are still often understaffed for patient volumes [12]. A robust interprofessional team may be most effective in implementing the Standards of Care across treatment settings and the cancer trajectory [32]. Utilizing screening tools to effectively match level of family risk to needed intervention, as demonstrated by the Pediatric Psychosocial Preventative Health Model, will allow for judicious use of psychosocial staffing resources [33,34].

In addition to staff, psychosocial providers indicated a desire for improved communication, opportunities to collaborate more effectively with others inside and across institutions, and a need for additional training and clinical supervision confirming previous research on the training needs of pediatric psycho-oncologists [35]. Participating in patient care rounds/medical team meetings and documenting in the electronic health record are recommended to facilitate communication, help medical staff better understand the role of psychosocial providers and improve integration of psychosocial providers into the pediatric oncology care settings [36].

*Implications for Psychosocial Providers:* This exploratory study is unique in that it explored how psychosocial providers approach treatment with children, caregivers, and siblings across the developmental and treatment trajectory. It is difficult to collect data about real-time clinical care due to nuance and complexity. This is a first step in identifying what interventions providers utilize most often. Some of the commonly reported interventions lack an evidence base for their use with the pediatric oncology population. An example of this is the often-endorsed use of apps. Conducting research on interventions that use technology (i.e., apps, telehealth, eHealth, etc.) has the potential to increase accessibility to patients and families across the treatment trajectory. Additionally, advocating for more psychosocial staff, resources, and training will allow sites to better implement and meet the Psychosocial Standards of Care. This includes making psychosocial oncology training more accessible across disciplines. 

It is important to note several limitations of the current study. The study population was confined to psychosocial providers who belong to one of the accessed professional organizations. Because there was no definite sample size, response rate could not be calculated. The study design was cross-sectional, so changes in services over time were not captured. All answers were based upon self-report. It was not possible to validate the reported interventions offered, or the specific dosing, frequency, and length of sessions. Delivery of reported interventions may vary based on patient geography and logistics around a patient’s medical care rather than symptom indications (e.g., travel distance, cost of travel, or insurance barriers). The survey did include an international population but was only offered in English, thereby excluding non-English speaking psychosocial providers. Because it was administered prior to the COVID-19 global pandemic, the survey did not include telehealth/virtual interventions. Future research should focus on strategies to increase accessibility, evaluate psychosocial intervention session dosing/frequency, institutional and programmatic support for psychotherapeutic services, and training for population-specific evidenced-based interventions and for translating science to practice.

## 5. Conclusions

Psychosocial providers offer a wide range of psychotherapeutic interventions to pediatric cancer patients and their families. Psychoeducation is the most utilized intervention throughout treatment. Cognitive behavioral therapy and mindfulness-based interventions are often used throughout the cancer trajectory. We found more limited use of oncology-specific evidence-based psychotherapeutic interventions. 

Implementation of the Standards requires a careful exploration of what is currently being provided in centers where children with cancer are being treated, which this study aimed to provide. The recently published Matrix and Guidelines provide the next step in identifying and implementing care consistent with the Standards [37]. Prior to their development, few guidelines offered recommendations for how to assess and manage psychosocial concerns of children with cancer [38]. Now, however, these Guidelines offer concrete action items for care centers and providers to improve provision of psychoeducation and anticipatory guidance, interventions for patients, caregivers, and siblings, communication of providers, and more. These efforts may help to close the identified gap between evidence-based care recommendations and what is provided in everyday practice to patients with cancer and their families.

## Figures and Tables

**Table 1:** Provider and institution characteristics.

**Variable**	**N**	**%**
*Degree (n = 242)*		
Bereavement Counselor	1	0.4
Child Life Specialist	12	5.0
Counselor	5	2.1
Nurse	9	3.7
Music Therapist	23	9.5
Psychiatrist	8	3.3
Psychologist	95	39.3
Social Worker	65	26.9
Other (Oncology, education, health)	24	9.9
*Professional Stage (n = 228)*		
Extern	1	0.4
Intern	3	1.2
Resident	2	0.8
Post-Doctoral Fellow	14	5.8
Early career licensed professional (<10 years from completion of highest degree)	89	36.8
Mid-career licensed professional (10–25 years from completion of highest degree)	78	32.2
Late-career licensed professional (>25 years from completion of highest degree)	34	14.0
Other	7	2.9
*Institution Type (check all that apply)*		
Academic medical center	114	47.1
Cancer-specific hospital	34	14.0
Community health center	1	0.4
Pediatric-specific hospital	139	57.4
Private practice	13	5.4
Other	14	5.8
*Work Setting (check all that apply)*		
Academic/research	82	33.9
Home care/hospice	8	3.3
Inpatient	180	74.4
Outpatient	165	68.2
Private practice	13	5.4
Other	9	3.7
*Country of practice (n = 227)*		
US	181	79.7
Europe (Belgium, France, Italy, Netherlands, Spain)	15	6.6
Canada	8	3.5
Middle East (Egypt, Israel, Pakistan, Turkey)	5	2.2
Africa (Nigeria, South Africa)	3	1.3
Scandinavia (Finland, Sweden)	3	1.3
Southeast Asia (India, Sri Lanka)	3	1.3
Australia	2	0.9
Brazil	2	0.9
Other	5	2.2
*Patients and psychosocial providers*	Mean	Range
New pediatric oncology patients seen each year	188.6	12–650
Number of social workers	4.1	0–27
Number of psychologists	2.4	0–20
Number of child life specialists	3.5	0–21

**Table 2:** Treatment population.

	**<5%**		**5–24%**		**25–49%**		**50–74%**		**75–100%**		**N/A**	
**Population**	**N**	**%**	**N**	**%**	**N**	**%**	**N**	**%**	**N**	**%**	**N**	**%**
Patients (0–12 years)	7	2.9	63	26.0	93	38.4	43	17.8	14	5.8	1	0.4
Patients (13–17 years)	7	2.9	74	30.6	110	45.5	18	7.4	10	4.1	1	0.4
Patients (18–25 years)	60	24.8	89	36.8	38	15.7	11	4.5	3	1.2	7	2.9
Caregivers	40	16.5	52	21.5	43	17.8	22	9.1	35	14.5	13	5.4
Siblings	108	44.6	52	21.5	9	3.7	4	1.7	6	2.5	18	7.4
Other (e.g., grandparents)	48	19.8	20	8.3	2	0.8	1	0.4	1	0.4	22	9.1

**Table 3:** Types of interventions offered throughout cancer trajectory.

**Intervention**	**Point in Cancer Trajectory**													
	**At Diagnosis**		**During Treatment**		**Maintenance/Transition Off-Treatment**		**Survivorship**		**Relapse**		**End of Life**		**Bereavement**	
	**N**	**%**	**N**	**%**	**N**	**%**	**N**	**%**	**N**	**%**	**N**	**%**	**N**	**%**
Anticipatory Guidance	99	40.9	102	42.1	88	36.4	68	28.1	83	34.3	80	14.1	47	19.4
Cognitive behavioral therapy (CBT)	65	26.9	102	42.1	92	38.0	76	31.4	68	28.1	45	9.5	27	11.2
Health promotion interventions	66	27.3	102	42.1	86	35.5	71	29.3	46	19.0	21	5.2	13	5.4
Mindfulness-based interventions	73	30.2	107	44.2	80	33.1	60	24.8	77	31.8	67	13.4	36	14.9
Pediatric-cancer specific interventions (e.g., Bright Ideas, Solving Cancer Competently)	41	16.9	78	32.2	46	19.0	35	14.5	23	9.5	9	3.7	6	2.5
Psychoeducation	115	47.5	118	48.8	101	41.7	86	35.5	93	38.4	84	12.8	61	25.2
Referrals to social support groups	72	29.8	92	38.0	68	28.	63	26.0	55	22.7	48	10.5	61	25.2
Supportive individual psychotherapy	82	33.9	94	38.8	84	34.7	71	29.3	84	34.7	83	14.8	62	25.6

**Table 4:** Clinical interventions used for each population.

**Intervention**	**Patients**		**Caregivers**		**Siblings**	
	**N**	**%**	**N**	**%**	**N**	**%**
Anticipatory Guidance	124	51.2	121	50.0	62	25.6
Cognitive behavioral therapy (CBT)	137	56.6	114	47.1	62	25.6
Health promotion interventions	145	59.9	105	43.4	47	19.4
Mindfulness-based interventions	140	57.9	101	41.7	55	22.7
Pediatric-cancer specific interventions (e.g., Bright Ideas, Solving Cancer Competently)	69	28.5	58	23.9	13	5.4
Psychoeducation	160	66.1	150	62.0	96	39.7
Referrals to social support groups	114	47.1	126	52.1	79	32.6
Supportive individual psychotherapy	132	54.5	105	43.4	61	25.2

**Table 5:** Apps recommended to patients and families.

**App Type**	**N**	**%**
Art	21	8.7
Adherence	26	10.7
Anxiety	48	19.8
Breathing/Relaxation	130	53.7
Coaching/Goal Setting	13	5.4
Guided imagery	78	32.2
Meditation	88	36.4
Mood tracking	28	11.6
Music	68	28.1
Pain	27	11.2
Other	8	3.3

**Table 6:** Educational and resource needs of psychosocial providers.

**Resource Type**	**Illustrative Quotes**
*Staff Resources*	
Additional staff (psychiatrists, art/music therapists, neuropsychologists, psychologists, social workers)	*“More pediatric psychologists available to help.”“More staff who support emotional help.*”
More time dedicated to providing direct care	*“Would love more time with patients and less time documenting.”*
Additional trainings including clinical supervision	*“More training about specific evidence-based interventions”* *“Clinical supervision!”*
Better work–life balance (time off, self-care resources)	*“Permission and support to engage in self-care.”*
*Communication and Collaboration*	
Strategies to better integrate psychosocial services into care	*“I’d like to know ways to really integrate the Standards of Care into practice.”*
More collaboration and communication between staff	*“More opportunities to collaborate across disciplines.”* *“Improved communication between staff about family and patient psychosocial needs.”* *“Built-in collaboration with medical and social work team.”*
Increased role awareness and professional advocacy	*“Being a valued member of a team would help.”* *“How to better articulate what it is that I do to the medical providers.”*
*Family Resources*	
Community-based mental health services	*“I need more information about mental health resources in the community.”*
Family-friendly resources on psychosocial care	*“Short guide to help parents cope at diagnosis like a generic version of the Psychosocial Standards of Care.”*

## Data Availability

Data available upon request from the corresponding author.

## Supplementary Materials

The following are available online at https://www.mdpi.com/article/10.3390/children8111045/s1, Supplementary File S1: Psycho-Oncology Interventions Survey.
