# Supplementary material for: Utilization of Psychotherapeutic Interventions by Pediatric Psychosocial Providers
Source: Children (Basel). 2021 Nov 12;8(11):1045. doi: 10.3390/children8111045 (PMC8625037; doi:10.3390/children8111045)
Supplement: Supplementary file 1 [file children-08-01045-s001.zip › children-1449144-supplementary.pdf]

Psycho-Oncology Interventions Survey

Introduction:
The purpose of this survey is to understand and learn more about the types of clinical/ therapeutic interventions being used by professionals providing psychosocial services to children with cancer and their family members. This survey will further our field by helping to clarify the current use and implementation of interventions. There are no right or wrong answers to any of these questions. Your answers are anonymous and confidential.

We hope you will complete this survey, even if you only work with children or families with cancer a small portion of your time. For the purpose of this study, we define youth as children, adolescents, and young adults up to the age of 25. We would like to have as great a representation of providers caring for the psychosocial needs of children as possible. Therefore, if you work with other trainees or licensed professionals in your institution that are providing psychotherapeutic interventions to children with cancer and their families (e.g., social workers, child life specialists, psychologists, psychiatrists, nurses, trainees), please forward this link. If there are multiple providers of the same specialty, please encourage all of them to complete.

This survey was approved by the XXX IRB. It should take approximately 15 minutes to complete.

Those who complete the survey will have the option to be entered to win one of 4 $50 Amazon gift cards. No identifying information will be linked with your survey responses.

If you have questions about this survey, please contact XXX or XXX. Thank you for your help!

Do you consent to participating in this anonymous survey?
- Accept
- Decline

What is your degree in?
- Bereavement Counseling
- Child Life Specialist
- Counseling
- Nursing
- Psychiatry

- Psychology
- Social Work
- Other (Please specify)

Which best fits your current professional stage?
- Extern
- Intern
- Resident
- Post-doctoral Fellow
- Early career licensed professional (<10 years from completion of highest degree)
- Mid-career licensed professional (10-25 years from completion of highest degree)
- Late-career licensed professional (>25 years from completion of highest degree)
- Other

How long have you been providing care to children with cancer and their families?
- <1 year
- 1-3 years
- 4-6 years
- 7-9 years
- 10-15 years
- >15 years

How many new pediatric oncology patients are seen each year at your institution? ___

Which work setting best applies to you? Please check all that apply.
- Inpatient
- Outpatient
- Private practice
- Academic/research
- Home care/hospice
- Other (Please specify)

What type of institution/setting do you work at? Please check all that apply.
- Academic medical center
- Community health center
- Cancer-specific hospital
- Pediatric-specific hospital
- Private practice
- Other (Please specify)

In what country to you practice in?

- Australia
- Belgium
- Brazil
- Canada
- China
- Egypt
- France
- Germany
- Italy
- Japan
- Mexico
- Netherlands
- Portugal
- Russia
- Spain
- United Kingdom
- United States
- Other (Please specify)

How many social workers at your site provide services to pediatric oncology patients? _____

How many psychologists at your site provide services to pediatric oncology patients? _____

How many child life specialists at your site provide services to pediatric oncology patients? _____

Availability of psychiatrists?

- None
- Sometimes
- Usually
- Always

Where did you receive or hear about this survey? Please check all that apply.

- APOS newsletter
- APOS Peds SIG newsletter/listserv
- APOSW listserv
- Child Life Specialist listserv

- POPPI newsletter
- AACAP Psychiatry listserv
- SIOP
- Division 54 (Heme/Onc SIG listserv)
- Colleague

What percentage of your practice is treating the following populations (please check all that apply):

- Patients (0-12 years old)
  - <5%
  - 5-24%
  - 25-49%
  - 50-74%
  - 75-100%
  - N/A
- Patients (13-17 years old)
  - <5%
  - 5-24%
  - 25-49%
  - 50-74%
  - 75-100%
  - N/A
- Patients (18-25 years old)
  - <5%
  - 5-24%
  - 25-49%
  - 50-74%
  - 75-100%
  - N/A
- Parents
  - <5%
  - 5-24%
  - 25-49%
  - 50-74%
  - 75-100%
  - N/A
- Siblings
  - <5%
  - 5-24%
  - 25-49%

- 50-74%
- 75-100%
- N/A

- Other (e.g., grandparents)
  - <5%
  - 5-24%
  - 25-49%
  - 50-74%
  - 75-100%
  - N/A

Thinking specifically about your practice, which of the following clinical interventions do ***you use*** *for each of the populations listed? Please check all that apply.*

Used among:

	Patients	Siblings	Parents (Primary Caregiver)
Acceptance and commitment therapy			
Adversity-Belief-Consequence Framework (ABC)			
Anticipatory Guidance			
Art/Expressive therapy (e.g., drawing, art-making)			
Behavioral Parent Training			
Bibliotherapy			
Biofeedback			
Bright IDEAS			
Cellie Cancer Coping Kit			
Cognitive behavioral therapy (CBT)			
CBT-Insomnia			
CBT-Pain			
Coping Cat			

Electronic/ Online games (e.g., Remission)			
Family and Marital Therapy			
Health promotion interventions (e.g., exercise interventions, psychoeducation, interactive media forums, online apps)			
Hypnosis			
Medical adherence interventions (e.g., Re-Mission 2 online games, MEMSCap, MedMinder)			
Medical Play			
Mindfulness-based interventions			
Motivational Interviewing			
Narrative therapy			
Onco-STEP (internet-based intervention for off-treatment survivors)			
Play therapy			
Psychoeducation			
Referrals to social support groups (either in-person or online)			
Social skills training			
Solution Focused Brief Therapy			
Supportive individual psychotherapy			
Supportive group psychotherapy			

Surviving Cancer Competently: Intervention Program (SCCIP)			
Therapeutic board games (e.g., ShopTalk)			
Therapeutic workbooks (e.g., This is My World)			
Trauma-focused CBT			
Other (Please describe)			

Are there other populations you work with besides patients, siblings, and parents (primary caregivers)? If yes, please describe.

- (Open-ended question)

Below are all the interventions that you endorsed, regardless of the population (i.e., patients, siblings, parents). Please indicate the following for each: 1) the frequency that you use this intervention in practice; and 2) when during the cancer trajectory, the intervention is used.

- Frequency of use
    - Rarely
    - Occasionally
    - A moderate amount
    - A great deal
- Timing of the intervention. Please check all that apply.
    - At diagnosis
    - During treatment
    - Maintenance/Transition off-treatment
    - Survivorship
    - Relapse
    - End of Life
    - Bereavement

If you use or suggest apps to patients and families, which of the following types of apps do you recommend? Please check all that apply.

- Art
- Adherence
- Anxiety

- Breathing/Relaxation
- Coaching/Goal Setting
- Guided imagery
- Health promotion (i.e., sleep hygiene, physical activity, nutrition)
- Meditation
- Mood tracking
- Music
- Pain
- N/A
- Other

When you provide psychoeducation to patients and families, which of the following categories does the education fall under? Please check all that apply.

- Age-appropriate communication with patient
- Age-appropriate communication with sibling(s)
- Communication with partner
- Communication with extended family, friends
- Communication with medical team
- Coping and adjustment of patient
- Coping and adjustment of parent(s)
- Coping and adjustment of sibling(s)
- Developmental Norms
- Diagnosis
- Health promotion (e.g., sleep hygiene, physical activity, nutrition)
- Hierarchy of Medical System/Provider roles
- Information about hospital/unit/policies/supports
- Information preferences
- Medical adherence, compliance
- Mindfulness, meditation
- Parenting/Discipline
- Phases of Treatment
- Procedural Preparation
- School Re-Entry
- Self-Care
- Social skills
- Symptom Management
- N/A
- Other

Would additional clinical supervision would be helpful to your work?

- Yes
- No
- Maybe

Name three things that would improve your ability to do your job better:

- 1. [free-text box]
- 2. [free-text box]
- 3. [free-text box]

Based on your clinical work and experience, are there any areas you would like additional training or resources on?

- Counseling
- E-health and electronic interventions
- Evidence-based interventions
- Research
- Telehealth
- Other (Please specify)

In your experience, what population has been the most challenging to work with from an intervention and resource perspective?

- Adolescents and young adults
- Adherence (e.g., medications)
- End-of-life patients
- Immigrants
- LGBTQ
- Non-English speakers
- Young children (infants, toddlers)
- Other (please specify)

Please feel free to add additional comments regarding the types of interventions you have found most successful, innovative or creative in helping children and their families with cancer.

- (open-ended question)

Thank you for your time to take this survey. Your responses and comments are greatly appreciated.
